# Pregnancy-Onset Ulcerative Colitis in a Pediatric Patient Presenting With Altered Mental Status and Severe Anemia

**DOI:** 10.7759/cureus.26434

**Published:** 2022-06-29

**Authors:** Alda Huang, Gregory L Stone, Brian Gordon, Gina J Kim

**Affiliations:** 1 Department of Combined Internal Medicine and Pediatrics, Los Angeles County University of Southern California Medical Center, Los Angeles, USA; 2 Department of Pediatrics, University of Southern California Keck School of Medicine, Los Angeles, USA; 3 Department of Obstetrics and Gynecology, Los Angeles County University of Southern California Medical Center, Los Angeles, USA

**Keywords:** adolescent pregnancy, pediatrics and neonatology, inpatient pediatrics, pediatric intensive care unit(picu), high-risk pregnancy, inflammatory bowel disease, ulcerative colitis (uc)

## Abstract

Ulcerative colitis (UC) classically presents with abdominal pain, hematochezia, or diarrhea. However, it can present atypically in pediatric and pregnant patients, posing a diagnostic challenge. A healthy, 16-year-old primigravida presented at 18 weeks and six days of gestation with sudden-onset altered mental status and severe anemia. Hematochezia began about 12 hours after admission. She underwent extensive workup, leading to an endoscopic and histopathologic diagnosis of UC, and achieved prenatal remission with high-dose steroids and infliximab. Her pregnancy, however, was complicated by severe preeclampsia, and her child’s post-delivery course was medically complex from an unrelated etiology. Pregnancy-onset inflammatory bowel disease (IBD) in the pediatric population is an uncommon but important consideration. Early diagnosis, treatment, and counseling are vital to achieve results comparable to those of patients without IBD.

## Introduction

Ulcerative colitis (UC), a subset of inflammatory bowel disease (IBD), is characterized by mucosal inflammation limited to the colon and terminal ileum and classically presents with bloody diarrhea with periods of remission and exacerbation [1]. Pathogenesis is due to dysregulated antigen recognition and inflammation, resulting in damage to the intestinal epithelium [1]. While its etiology is unclear, the prevailing theory involves a deleterious interaction with intestinal microbiota due to a genetic predisposition [1].

The prevalence of UC is highest in North America and Europe, which ranges from 8 to 238 per 100,000 people [2]. While the incidence of UC is highest in those aged 15-30 years [1], it has been increasing in the pediatric population, especially in those under five years, likely due to improvement in diagnostic techniques and the recognition that pediatric patients often present atypically [3,4]. Another challenging population in the diagnosis and management of UC is pregnant women. While pregnancy does not seem to be a trigger for disease exacerbation, those with active disease during pregnancy may be more resistant to treatment [5]. UC has also been associated with preterm delivery and low birth weight, especially in cases where the disease is more active during pregnancy [5].

We report a novel initial presentation of UC in a pregnant pediatric patient during her second trimester of pregnancy. Given that the pediatric pregnant patient with UC is a unique and poorly understood clinical entity, this case provides insight into how the interaction between age and pregnancy affects the natural course of UC and pregnancy outcomes in these patients.

## Case presentation

A previously healthy 16-year-old gravida 1 para 0 (gestational age 18 weeks, 6 days) female was admitted to the pediatric intensive care unit (PICU) for management of severe anemia and acute encephalopathy.

The patient was in her usual state of health until around noon on the day of admission, at which time she had a sudden-onset headache and abdominal pain. She reportedly took one 325 mg tablet of acetaminophen for symptomatic relief. Shortly after, her family found her with altered behavior for which she was brought to the hospital. On arrival, the patient was awake but oriented to name only and not following commands. The patient desired her pregnancy and had established prenatal care appropriately. A week prior to presentation, routine hemoglobin obtained by her prenatal care provider was 9.8 g/dL.

At presentation, she was afebrile, tachycardic (heart rate of 147 beats per minute), normotensive (blood pressure 117/63 mmHg), and oxygenating well on room air. Initial labs were remarkable for a hemoglobin of 3.5 g/dL and a hematocrit of 11.1%. Her white blood cell count was normal but showed neutrophilic predominance. An extensive workup for acute encephalopathy was also initiated. The electrolytes and glucose were non-contributory, and the patient had a normal thyroid-stimulating hormone level. Heavy metal poisoning, which can cause encephalopathy, was ruled out by normal serum levels. The family stated that during the three weeks prior to presentation, the patient was having a depressed mood in response to an identified stressor but had been coping well with family support, and there was no elicited history of prior or current substance abuse, which was confirmed by the patient later in her hospital course. In addition, her urine toxicology was negative, as were her serum salicylate, acetaminophen, and alcohol levels. Computed tomography and magnetic resonance imaging of the brain were unremarkable.

She was given 3 units of packed red blood cells (PRBCs) during the first 36 hours of admission with an appropriate hemoglobin response of 9.4 g/dL. Her encephalopathy also gradually improved and completely resolved approximately 40 hours after the initial presentation. Around 40 hours after initial presentation, the patient had a dark-red bloody discharge with tissue-like material, initially thought to be vaginal in origin. A pelvic exam showed a closed cervix with no blood in the vagina, and an obstetric ultrasound demonstrated a viable intrauterine gestation. Further examination revealed the source of the bloody discharge to be rectal. Repeat labs indicated acute blood loss once again with a hemoglobin drop to 7.0 g/dL. The patient denied any diarrhea, melena, hematochezia, or vaginal discharge prior to admission. She continued to have bloody bowel movements 12 to 16 times per day over the next five hospital days, requiring 4 additional units of PRBCs during her hospital course to maintain her hemoglobin above 9 g/dL.

**Figure 1:**
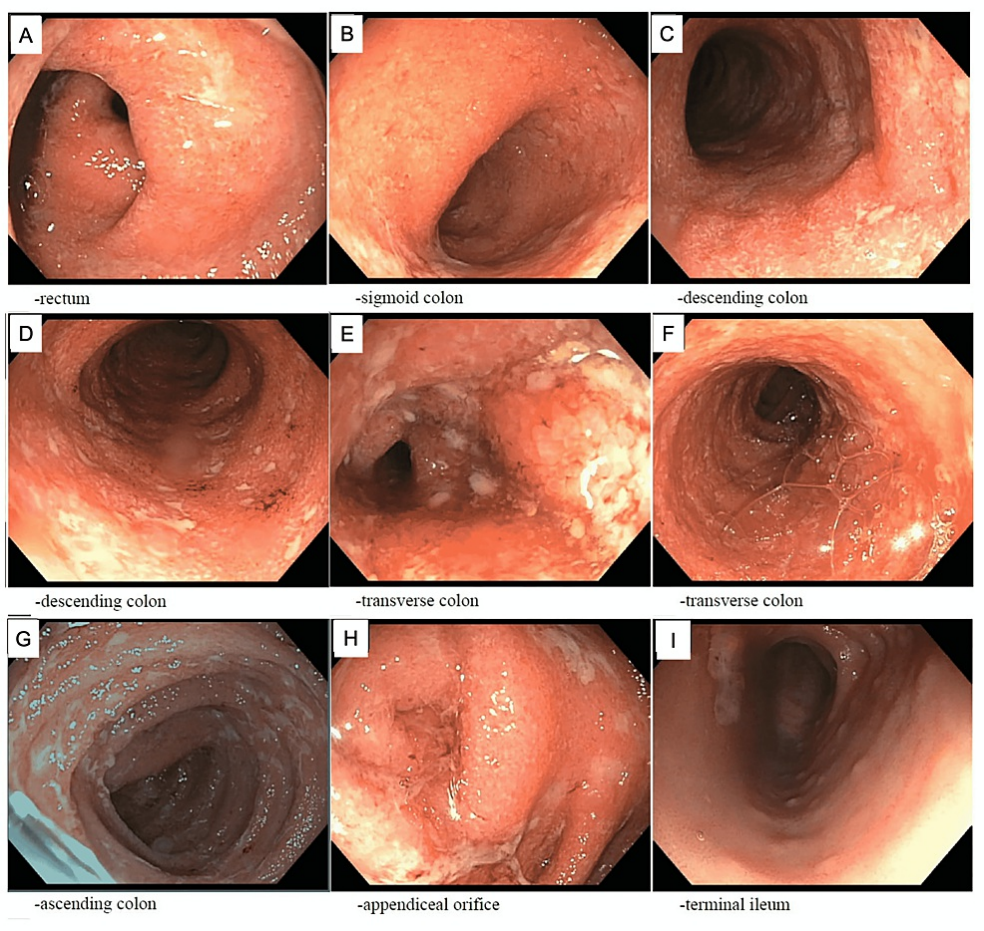
Colonoscopy findings. Diffuse erythema, edema, loss of vasculature, and ulceration was noted in the entire colon: the rectum (A), sigmoid colon (B), descending colon (C-D), transverse colon (E-F), and ascending colon (G). The appendiceal orifice was also involved (H). Blunting of the villi of her terminal ileum was without erythema or ulceration (I). Mild, active idiopathic inflammatory disease was noted in the descending colon and sigmoid colon (B-D). Moderately active disease was present on surgical pathology in the rectum (A).

Given the presence of bloody diarrhea in the setting of acute encephalopathy, tachycardia, and neutrophilic predominance of white blood cells, the patient was started on empiric broad-spectrum antibiotics for suspected infectious colitis, a common cause of bloody diarrhea in pediatrics. Stool tests for Shiga toxin antigen, Clostridium difficile toxin, and ova and parasites were negative. The patient also had negative QuantiFERON, Epstein-Barr virus, and Cytomegalovirus serologies. Urine, blood, and stool cultures were obtained on admission and showed no growth, prompting the discontinuation of antibiotic therapy. At this time, suspicion of IBD became higher, supported by the presence of elevated serum inflammatory markers and elevated fecal calprotectin in the absence of identifiable infection. The patient underwent esophagogastroduodenoscopy (EGD) and colonoscopy with biopsy for the assessment of active gastroenteric bleeding and inflammation. EGD was unremarkable, but colonoscopy demonstrated diffuse erythema, edema, loss of vasculature, ulceration from the rectum to the cecum, and blunting of the villi of the terminal ileum without erythema or ulceration (Figure 1).

**Figure 2:**
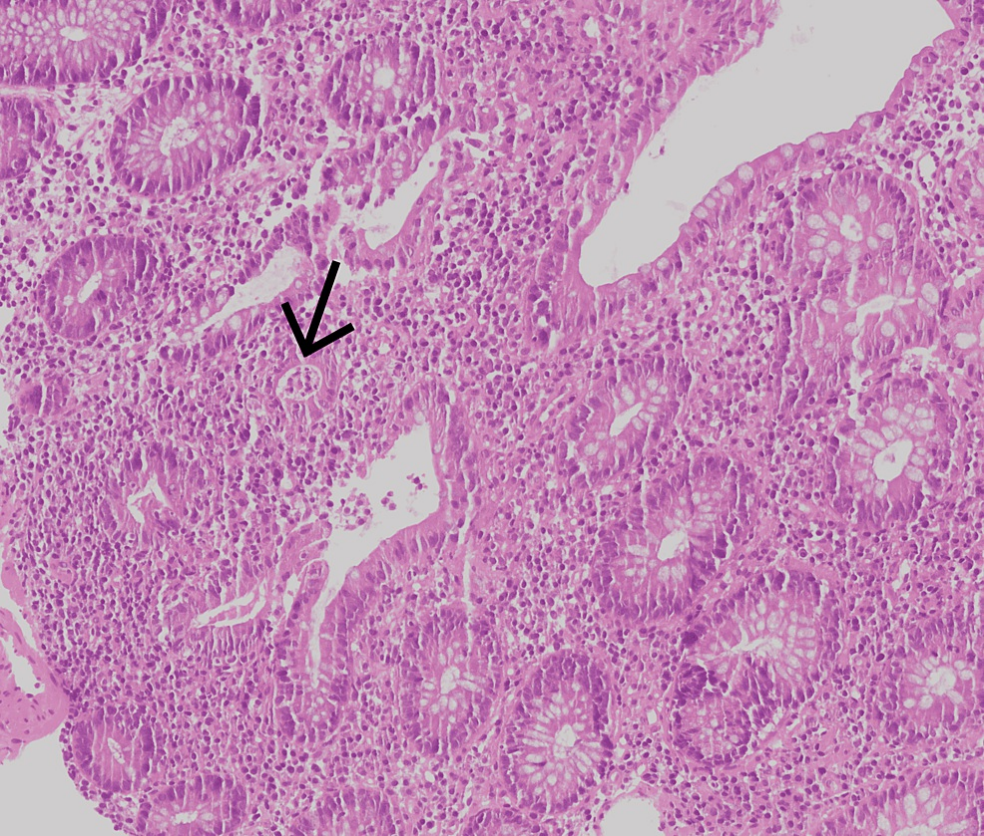
Histopathologic findings. The patient's colon biopsy showed moderate inflammatory changes and crypt abscesses (arrow), which are classic for ulcerative colitis.

Biopsy findings of the left colon were concerning for IBD (Figure 2).

The diagnosis of UC was made, and high-dose methylprednisolone was initiated. Within 48 hours of steroid initiation, the frequency of stools lessened to four to five stools daily with less blood. However, given the persistence of frank blood in stools after several days of steroids, her first infliximab infusion (dosed at 5 mg/kg) was initiated. Following her first infliximab infusion, her stools were more formed, non-bloody, and occurred one to two times daily, indicating resolution of the UC flare. She was transitioned to an oral steroid taper and discharged on hospital day 18, with a plan to continue infliximab infusions every two weeks. Three days later, at her outpatient GI follow-up, the patient endorsed that her stooling pattern had returned to baseline. She continued to do well with regular infliximab infusions every six weeks until 28 weeks of gestation. It was held at the beginning of her third trimester, as the placental transfer is highest at that time, and resumed on postpartum day 1. Her pregnancy was complicated by pregnancy-induced hypertension at 34 weeks and 6 days of gestation, but fetal growth was unremarkable on serial ultrasonography. She eventually developed severe preeclampsia with severe features and had an uncomplicated vaginal delivery of a baby boy at 36 weeks and 1 day with a birth weight of 2275 g (15th percentile). The patient was discharged without incident on postpartum day 3 and has not had any recurrence of her UC to date.

The baby initially required invasive mechanical ventilation but was extubated to room air within 24 hours. His hospital course was complicated by hypoxic-ischemic encephalopathy; subgaleal, cephalo-, and subdural hematomas; Escherichia coli meningitis and pneumonia; poor feeding; and hypotonia, all later attributed to Pompe’s disease, a rare inherited disorder caused by mutations in a gene that makes the enzyme acid alpha-glucosidase, detected on newborn screens. He was discharged home after 29 days in the neonatal intensive care unit and is being followed closely by primary care and subspecialists. At his two-month follow-up, the baby was growing appropriately and meeting developmental milestones.

## Discussion

Pregnancy-onset IBD (POIBD) is an uncommon clinical entity described in small retrospective studies. There is a paucity of literature regarding POBID in the pediatric population since most studies do not include pregnant women under the age of 18 years. Given that over 30% of women are sexually active by the age of 16 with a pregnancy rate of >100 per 1000 females and increases with age [6], IBD in the pregnant adolescent is an important clinical entity to diagnose and manage.

POIBD can be diagnosed during any trimester and the first post-partum year. A retrospective study from Massachusetts showed that there was a slightly higher symptom onset during the first trimester, patients were more likely to have UC than non-pregnant IBD patients, and those with UC appeared to be more symptomatic and had more hospitalizations than those with Crohn’s [5,7]. Mean gestational age and neonatal birth weight were similar between POIBD and non-POIBD patients [7]. Uncontrolled IBD during pregnancy has been associated with preterm labor and low birth weight [8]. Since treatment adherence has been shown to be low in the pediatric IBD population [9], it is imperative that physicians counsel patients and families about the importance of treatment, as lower disease activity during pregnancy is associated with better outcomes [8].

Our patient presented atypically in that she had profound anemia and encephalopathy that preceded hematochezia. Although anemia can be seen in IBD, the degree of anemia in our patient in the absence of a history of hematochezia was striking. It is possible that the patient had been accumulating bloody stool intra-luminally but was unable to defecate until she had experienced significant colonic bleeding due to a compressive effect of the gravid uterus on the colon [10]. The patient’s encephalopathy may have been due to psychosocial stressors or her severe, acute anemia as a result of her UC. It is also possible that the patient had concomitant toxic ingestion that could not be measured with our institution’s assays.

Our patient improved following administration of glucocorticoids followed by anti-tumor necrosis factor (TNF)-alpha antibody therapy with infliximab. For both pediatric and pregnant patients, glucocorticoids and biologics are recommended for the treatment of acute colitis and should not influence the decision to breastfeed [11,12]. Furthermore, with the exception of methotrexate, pregnant women should continue their medication throughout pregnancy, including azathioprine, and preconception counseling is recommended to minimize disease activity at conception and throughout pregnancy [12]. A prospective study of Janssen Biologics’ safety data on infliximab-exposed pregnancies since 1998 revealed no increase in the rate of adverse pregnancy and infant outcomes compared to the general population, regardless of whether infliximab exposure occurred during the first or third trimester [12]. However, since the effect of infliximab on the neonate’s immune system is unknown and the rate of placental crossing of infliximab is highest during the third trimester, it is recommended to be held in the third trimester but can be safely restarted immediately postpartum [12]. The adverse perinatal outcomes observed in this case were therefore unlikely to have been due to maternal UC. Likewise, the patient’s preeclampsia is unlikely to be related to her UC or her treatment course, given she maintained remission of her UC after initiation of treatment.

## Conclusions

In conclusion, POIBD in pediatrics is an uncommon but important entity to consider. Because it can present atypically in this population, and adolescents are less likely to adhere to treatment, these patients are most vulnerable to adverse events. Uncontrolled IBD during pregnancy leads to poor neonatal outcomes, whereas remission throughout pregnancy leads to results comparable to non-IBD patients. Therefore, early diagnosis, treatment, and counseling are vital to prevent significant morbidity.
